# Sugar-sweetened beverage consumption predicts metabolic associated fatty liver disease in patients with type 2 diabetes mellitus

**DOI:** 10.3389/fendo.2025.1651370

**Published:** 2025-10-01

**Authors:** Zhenjun Yu, Mengdie Chen, Shicheng Gu, Chaohui Wang, Ping Feng, Gang Lin

**Affiliations:** ^1^ Department of Gastroenterology, Taizhou Central Hospital (Taizhou University Hospital), Taizhou, Zhejiang, China; ^2^ Department of Endocrinology, Taizhou Central Hospital (Taizhou University Hospital), Taizhou, Zhejiang, China

**Keywords:** type 2 diabetes mellitus, metabolic associated fatty liver disease, glycometabolism, sugar-sweetened beverage, risk prediction

## Abstract

**Background:**

Metabolic associated fatty liver disease (MAFLD) is a leading cause of chronic liver disease worldwide, with heightened prevalence and progression risks in individuals with type 2 diabetes mellitus (T2DM). Emerging evidence suggests dietary factors, particularly sugar-sweetened beverage (SSB) consumption, may exacerbate metabolic dysregulation, yet this relationship remains underexplored in MAFLD populations.

**Method:**

We enrolled 3,305 T2DM patients from Taizhou University Hospital, classifying them into MAFLD and non-MAFLD groups via liver ultrasonography. SSB consumption was quantified as weekly intake. Clinical parameters and SSB consumption were analyzed using logistic regression. External validation leveraged NHANES data, focusing on total sugar intake and surrogate markers.

**Results:**

MAFLD patients exhibited significantly higher BMI, waist/hip ratios, and SSB consumption than non-MAFLD counterparts (p<0.001). SSB consumption emerged as an independent MAFLD risk factor, with dose-dependent escalation in MAFLD odds. The MAFLD model based on glycometabolism (MMBG), integrating SSB consumption, C-peptide, and glucose, outperformed traditional indices, such as TyG, VAI, and AIP, achieving superior AUC (0.712 vs. 0.631–0.666), enhanced clinical utility and higher Brier scores (p<0.05, respectively). NHANES validation confirmed BMI, central obesity, hyperglycemia, and sugar intake as MAFLD predictors.

**Conclusion:**

SSB consumption independently predicts MAFLD risk in T2DM patients, with synergistic effects from dysregulated glycometabolism. The MMBG model, incorporating SSB consumption and glycometabolic parameters, offers a robust tool for early MAFLD risk identification and personalized interventions.

## Introduction

Metabolic associated fatty liver disease (MAFLD) (1) stands as the most prevalent chronic liver disease globally, with the latest statistics revealing that approximately one- (2) and one-quarter of adolescents (3) are affected, thus ranking it among the foremost non-communicable diseases. Notably, the prevalence of MAFLD exhibits ethnic disparities, with Hispanics having the highest rate of 51.4% and African Americans the lowest at 21.5% (4). A comprehensive meta-analysis conducted in Asia reveals that the overall prevalence of MAFLD among Asian adults is 29.6% (4). By 2030, China is anticipated to witness a staggering number of 315 million MAFLD patients, positioning it as the country with the fastest growth (5).

MAFLD is a chronic and progressive disease characterized by excessive accumulation of fat in the liver, constituting 5% or more of the liver’s weight (6). The latest diagnostic criteria for MAFLD, released in 2020, categorize the condition into three groups: type 2 diabetes mellitus (T2DM), obesity, and non-obesity without T2DM (1). The development of MAFLD are intimately linked to metabolic disorders and insulin resistance, resulting in a significantly higher prevalence among T2DM patients, ranging from 60 to 75% (7, 8). Notably, T2DM and MAFLD share insulin resistance and systemic hyperinsulinemia as common pathophysiological mechanisms, which not only heighten the risk of mutual exacerbation but also influence the natural course (9). Specifically, T2DM exacerbate MAFLD by promoting hepatitis or fibrosis, whereas MAFLD worsen the natural progression of diabetic complications, including microvascular and macrovascular issues, in T2DM patients (10). Furthermore, MAFLD is associated with an increased incidence and mortality rate of extrahepatic diseases, such as cardiovascular and cerebrovascular diseases, as well as chronic kidney disease (11). Increasing evidence suggests that MAFLD actively participates in the pathogenesis of these complications, rather than serving as a metabolic marker (12).

MAFLD can evolve from simple steatosis to Metabolic steatohepatitis (MASH), fibrosis, cirrhosis, and ultimately hepatocellular carcinoma (HCC) (13). Given the absence or inconsistency of MAFLD guidelines, coupled with the asymptomatic nature in early stage, it is not uncommon for diabetic patients to receive delayed diagnoses of NASH, cirrhosis, or HCC (13). T2DM poses a significant global public health burden, accounting for approximately 90-95% of all diabetes (14). Over the past four decades, the global prevalence of diabetes among adults has quadrupled, rising from 108 million in 1980 to 463 million in 2019 (15). Consequently, it is imperative for the T2DM population to undergo systematic non-invasive testing. Assessing patients’ metabolic fatty liver conditions, based on practical anthropometric and biological parameters, along with potential glucose monitoring, holds significant importance for accurate disease assessment and tailored interventional strategies.

While obesity and dysglycemia are established MAFLD drivers, the role of dietary sugar particularly sugar-sweetened beverages (SSB) remains contentious. SSB contribute >40% of added sugar intake globally, promoting hepatic *de novo* lipogenesis and insulin resistance (16). Although SSB consumption correlates with non-alcoholic fatty liver disease (NAFLD) (17), MAFLD’s distinct diagnostic framework, encompassing concurrent metabolic and liver disorders (18), necessitates dedicated investigation. Notably, no prior studies have examined SSB-MAFLD associations in T2DM populations, a high-risk cohort with compounded metabolic vulnerabilities.

## Materials and methods

### Subjects

Between June 2020 and May 2024, a total of 3,776 patients with diabetes were screened at the Metabolic Management Center (MMC) of Taizhou Central Hospital (Taizhou University Hospital). The diagnosis of T2DM was made in accordance with the Chinese T2DM guidelines (19). The diagnostic criteria for MAFLD encompassed imaging evidence of hepatic steatosis, coupled with the presence of any one of the following three additional criteria: overweight/obesity, T2DM, or metabolic dysfunctions (1). All patients underwent ultrasound examination for the diagnosis of hepatic steatosis. The diagnosis was confirmed when two of the following three criteria were met: diffuse enhancement of liver near-field ultrasound echoes, liver echoes stronger than kidney echoes, blurred vascular distribution, and gradual attenuation of far-field ultrasound echoes.

Exclusion criteria: 1) Lack of crucial information such as age, gender, or ultrasound examination; 2) Presence of Cushing’s syndrome, receipt of total parenteral nutrition, and administration of medications that may induce specific fatty liver conditions, including amiodarone, valproic glucocorticoids, methotrexate, etc. 3) Diagnosis of other types of diabetes; 4) Malignant tumors; 5) Pregnant and lactating women.

**Figure 1:**
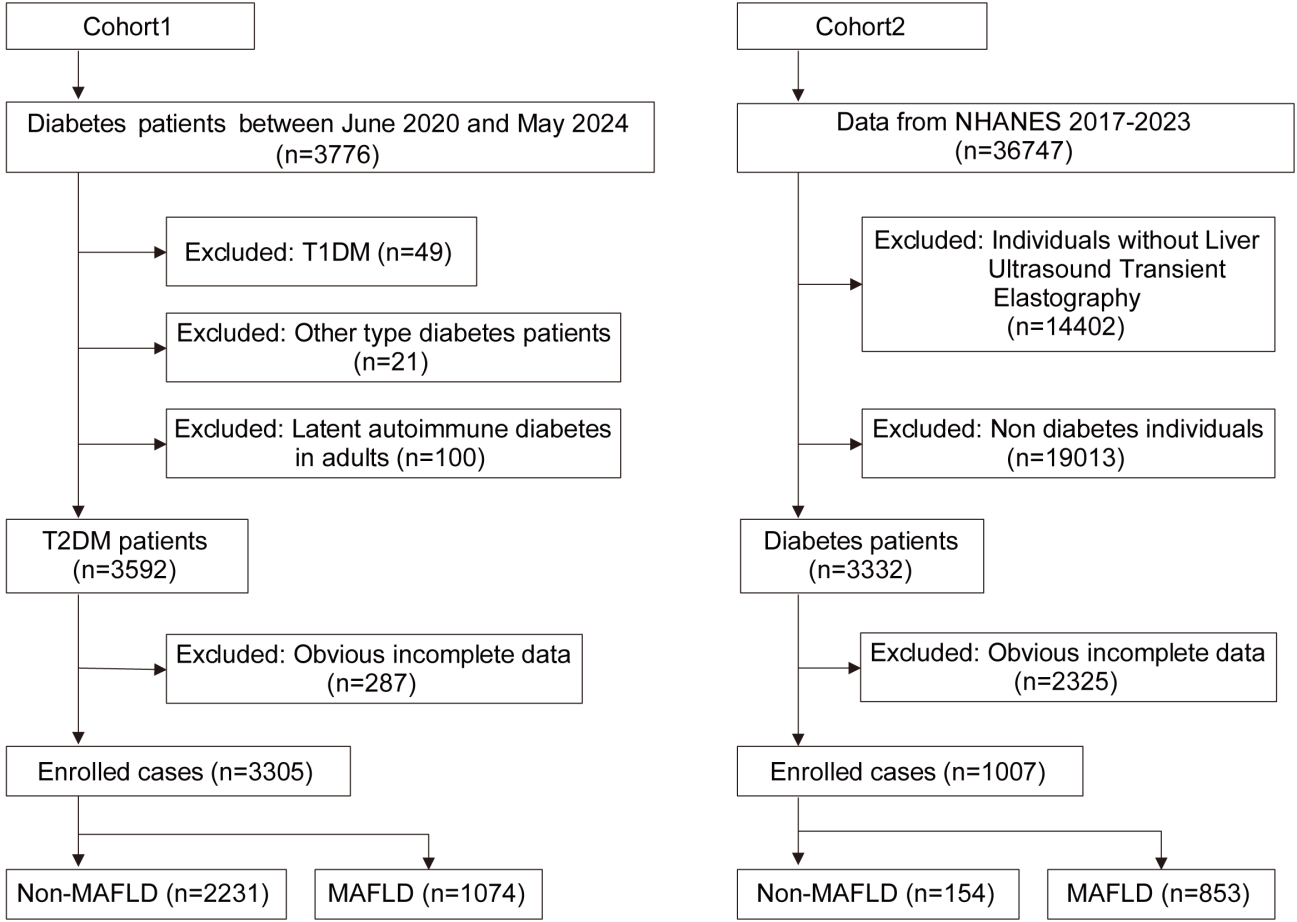
Flow chart of enrolled cases. T2DM, Type 2 diabetes mellitus; T1DM, Type 1 diabetes mellitus; MAFLD, Metabolic associated fatty liver disease.

Patients with type 1 diabetes (n=49), suspected late-onset autoimmune diabetes in adults with positive GAD antibodies (n=100), or other type of diabetes (n=35), as well as individuals with significant information gaps or comorbid conditions (n=287), were excluded from the study. A total of 3,305 individuals were enrolled in this study. Of these, 1,074 patients with confirmed fatty liver disease comprised the MAFLD group, while 2231 patients without fatty liver disease constituted the non-MAFLD group (**Figure 1**). The study protocol was approved by the ethics committee of the participating hospital, and all participants provided written informed consent.

### General information and clinical parameters

Comprehensive clinical data were collected from all enrolled patients, encompassing demographics such as age and gender, lifestyle habits including drinking and smoking histories, past medical conditions, educational background, and household annual income. Additionally, dietary intakes of vegetables, fruits, fish, beans, salt, and SSB, as well as sleep patterns, were meticulously recorded. The specific volume of one bottle of sugar-sweetened beverage was defined as 500ml. Sugar-sweetened beverages refer to those with added sugars (e.g., sucrose, high-fructose corn syrup, glucose, fructose) during production or preparation, or naturally high-sugar liquid foods primarily consumed as beverages, excluding ultra-processed beverages containing sweeteners and chemical additives. For drinking history, regular drinking was categorized as consuming at least one standard alcoholic beverage per week for a minimum of six months, while infrequent drinking fell short of this criterion. Similarly, in smoking history, regular smoking was defined as smoking more than 10 cigarettes weekly for at least six months, with anything less considered occasional smoking.

On the first day of admission, patients’ anthropometric measurements were taken in a fasting state, including height, weight, Body Mass Index (BMI), head, neck, waist, and hip circumference. On the second day, fasting blood samples were collected early in the morning for the unified assessment of glucose (GLU), C-peptide, serum creatinine (Cr), and glycosylated hemoglobin (HbA1C). Postprandial GLU and C-peptide levels were also evaluated two hours after a meal. Furthermore, urine samples were collected in a fasting state for the uniform analysis of urine protein, sugar, ketones, and creatinine.

### NHANES database

The NHANES database served as the validation set for our study. NHANES, a public database, employs a cross-sectional, stratified, and multi-stage probability design to capture a representative sample of the civilian, non-hospitalized population in the United States. This database comprises comprehensive survey data, encompassing questionnaires, demographic information, laboratory test results, and physical examination details. The research protocol adhered to ethical standards and received approval from the Research Ethics Review Committee of the National Health Center. All participants provided written informed consent prior to their involvement. Detailed information on the study design and survey procedures is accessible online (https://wwwn.cdc.gov/nchs/nhanes/).

We retrieved population data from the NHANES database, focusing on individuals from the testing cycles between 2017 and 2023. Out of the total 36,747 individuals extracted, we excluded 14,402 individuals due to the absence of liver ultrasound data, 19,013 non-diabetics, and 2,325 individuals with incomplete data. Consequently, a cohort of 1,007 individuals was established. Based on ultrasound findings, 853 cases with CAP value exceeding 248 were categorized as the MAFLD group, while remaining 154 cases comprised the non-MAFLD group. The wtint2yr data was utilized to analyze the weight information of cases. (**Figure 1**)

### Statistical analysis

Statistical analysis was conducted using SPSS 22.0 (IBM Corp., Armonk, New York, USA), R (version 4.4.1, Foundation for Statistical Computing, Vienna, Austria, https://www.R-project.org), and the nhanesR package. Multiple imputation of data was accomplished utilizing the MICE package. For measurement data, t-tests were applied for normally distributed data, while rank sum tests were employed for skewed distributions. The results were presented as Mean±SD or median (interquartile range). χ2 tests were utilized for the analysis of count data, and all data were represented as proportions. Univariate and multivariate logistic regression analyses were performed to explore the association between various parameters and the MAFLD. The rcssci package facilitated the creation of restricted cubic splines (RCS). Receiver operating characteristic (ROC) curves and Decision Curve Analysis (DCA) were utilized to assess the predictive value of different indicators. The DeLong test was employed to compare the areas under the ROC curves (AUC). A p-value of less than 0.05 was considered statistically significant.

## Results

### General characteristics of T2DM

A total of 3,305 patients with T2DM were enrolled in the study, with 1,074 (28.4%) belonging to the MAFLD group. To address missing data, the MICE package was utilized for multiple imputation (**Supplementary Figure S1**). Notably, the proportion of males in the MAFLD group (73.8%) was significantly higher compared to the non-MAFLD group (69.2%) (p<0.01). Furthermore, the mean age of patients in the MAFLD group (49.0±13.0 years) was significantly lower than that of the non-MAFLD group (52.3±11.8 years). (p<0.001, respectively) (**Table 1**). An analysis of patient characteristics revealed that the MAFLD group exhibited significantly higher BMI, head, neck, waist, and hip circumference compared to the non-MAFLD group (p<0.001, respectively) (**Table 1**). These findings underscore the distinct bodily characteristics of the MAFLD group, particularly their elevated BMI and waist circumference.

**Table 1:** General characteristics of enrolled T2DM patients.

Variables		Non-MAFLD (n=2231)	MAFLD (n=1074)	t/χ2/Z value	*p* value
Age (years)		52.3±11.8	49.0±13.0	7.052	<0.001
Gender (Male)		1543(69.2%)	793 (73.8%)	7.644	0.006
BMI (kg/m^2^)		24.52±3.66	26.82±3.69	16.873	<0.001
Head circumference (cm)		55.92±2.40	56.67±2.24	8.564	<0.001
Neck circumference (cm)		36.62±3.64	38.40±3.42	13.678	<0.001
Waistline (cm^2^)		87.38±9.76	93.52±9.11	17.738	<0.001
Hip circumference (cm^2^)		93.69±6.97	97.01±6.88	12.867	<0.001
Education (a high school education or higher)		733(32.9%)	465(43.3%)	34.200	<0.001
Full time job		1424(63.8%)	753(70.1%)	12.734	<0.001
Annual household income (¥)	<10000	50(2.2%)	16(1.5%)	2.720	0.007
	10000-30000	117(5.2%)	43(4.0%)		
	30000-100000	497(22.3%)	224(20.9%)		
	100000-300000	1030(46.2%)	494(46.0%)		
	>300000	537(24.1%)	297(27.7%)		
Smoking	None	1560(69.9%)	722(67.2%)	1.523	0.128
	Infrequent	95(4.3%)	52(4.8%)		
	Regular	576(25.8%)	300(27.9%)		
Drinking (alcohol)	None	1445(64.8%)	665(61.9%)	1.548	0.122
	Infrequent	643(28.8%)	336(31.3%)		
	Regular	143(6.4%)	73(6.8%)		
Vegetables (per day)	<200g	531(23.8%)	312(29.1%)	3.957	<0.001
	200-400g	1102(49.4%)	529(49.3%)		
	400-600g	566(25.4%)	224(20.9%)		
	>600g	32(14.3%)	9(8.4%)		
Fruit (per day)	<200g	1552(69.6%)	696(64.8%)	3.042	0.002
	200-400g	542(24.3%)	281(26.2%)		
	400-600g	112(5.0%)	81(7.5%)		
	>600g	25(1.1%)	16(1.5%)		
Fish (per day)	<1 time	430(19.3%)	221(20.6%)	0.850	0.395
	>2 times, <100g/time	350(15.7%)	169(15.7%)		
	>2 times, >100g/time	1451(65.0%)	684(63.7%)		
Bean (per day)	<100g	1310(58.7%)	647(60.2%)	0.522	0.602
	100-250g	823(36.9%)	367(34.2%)		
	250-400g	76(3.4%)	47(4.4%)		
	>400g	22(1.0%)	13(1.2%)		
Salt (per day)	<4g	113(5.1%)	46(4.3%)	0.925	0.355
	4-6g	633(28.4%)	302(28.1%)		
	6-8g	1073(48.1%)	515(48.0%)		
	>8g	412(18.5%)	211(19.6%)		
Sugar-Sweetened Beverage (per week)	None or < 1 bottle	1804(80.1%)	750(69.8%)	7.302	<0.001
	1–2 bottles	194(8.7%)	121(11.3%)		
	3–4 bottles	79(3.5%)	71(6.6%)		
	More than 5 bottles	154(6.9%)	132(12.3%)		
Sleep	Good	1781(79.8%)	837(77.9%)	1.255	0.209
	Poor	442(19.8%)	233(21.7%)		
	Drug dependence	8(0.4%)	4(0.4%)		

T2DM, Type 2 diabetes mellitus; MAFLD, Metabolic associated fatty liver disease; BMI, Body Mass Index.

The analysis of patients’ employment and income statuses revealed that within the MAFLD group, 465 cases (43.3%) with high school education or higher, 753 (70.1%) were engaged in full-time employment, and 297 (27.7%) had an annual income exceeding 300,000 yuan. These percentages were significantly higher than those observed in the non-MAFLD group, where 733 patients (32.9%) had a high school education or higher, 1424 (63.8%) were employed full-time, and 537 (24.1%) earned an annual income exceeding 300,000 yuan (p<0.01, respectively) (**Table 1**). In terms of dietary habits, patients in the MAFLD group exhibited significantly lower average daily vegetable intake compared to the non-MAFLD group, whereas their average daily fruit intake was notably higher. No significant differences were observed in terms of daily intake of beans, fish, salt, as well as the alcohol consumption, smoking habits, and sleep patterns. Regarding SSB, 324 cases (30.2%) in the MAFLD group reported consuming more than one sugary drink per week, a significantly higher rate than the 427 patients (19.1%) in the non-MAFLD group (p<0.001). Therefore, T2DM with MAFLD tend to have a higher proportion of individuals with higher education and income levels, the higher intake of SSB or fruits may increase the risk of developing MAFLD.

**Figure 2:**
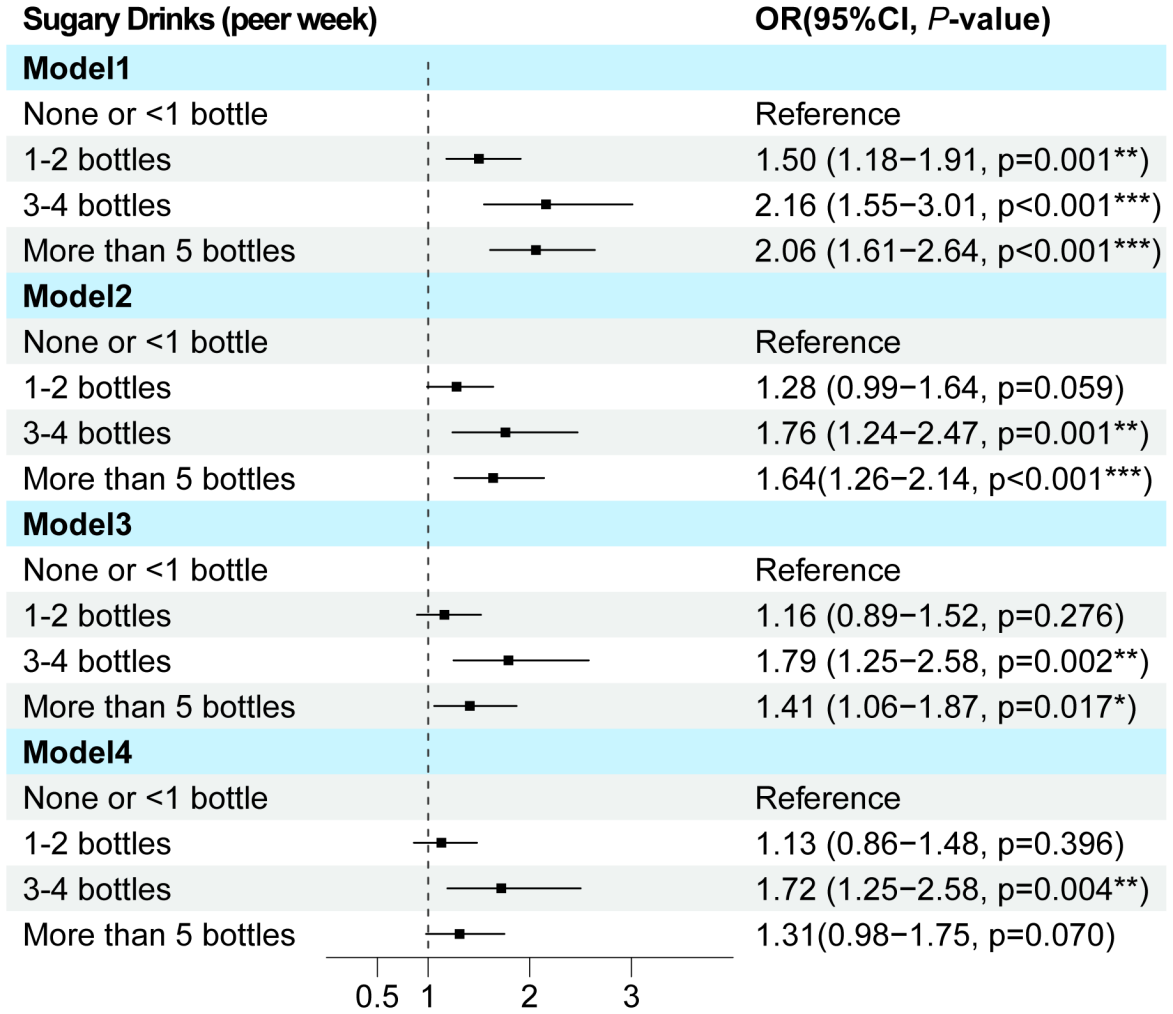
The multi-model logistic regression analysis for MAFLD exploring consumption of SSB in T2DM. Model 1 focused on a single-factor logistic regression for SSB. Model 2 adjusted for the confounding factors of age and gender. Model 3 further incorporated BMI, waist circumference, and education level into the lastly. Model 4 adjusted for all pertinent general characteristic parameters associated with MAFLD. T2DM, Type 2 diabetes mellitus; MAFLD, Metabolic associated fatty liver disease; OR, Odds ratio.

Univariate and multivariate logistic regression analyses demonstrated that gender, BMI, neck, waist, hip circumference, education, and SSB consumption were independent predictors of MAFLD (**Supplementary Table S1**; **Supplementary Figure S2**). To further investigate the impact of SSB on MAFLD, we constructed another three models: Model 2 adjusted for age and gender, Model 3 adjusted for age, gender, BMI, waist circumference, and education level, and Model 4 adjusted for all other factors. Notably, across all models, SSB consumption emerged as an independent risk factor for MAFLD, with a clear trend indicating that increased beverage intake is associated with a heightened risk of MAFLD in T2DM (p value for trend <0.001) (**Supplementary Table S2**; **Figure 2**).

### Abnormal glycometabolism correlated with MAFLD

The fasting and postprandial GLU, C-peptide, HbA1c levels, as well as creatinine, urine protein were individually assayed to assess glycometabolism in T2DM patients. The results revealed significantly elevated levels in the MAFLD group compared to the non-MAFLD group, specifically: postprandial GLU (14.26±4.88 mmol/L vs. 13.60±4.89 mmol/L), fasting C-peptide (2.14±1.24 mmol/L vs. 1.72±1.13 mmol/L), postprandial C-peptide (4.21±2.79 vs. 3.59±2.56), HbA1c (9.77±2.34 vs. 9.47±2.60), and urine creatinine (12164.56±6741.25 vs. 11219.40±6623.04) (p<0.01, respectively). No significant differences were observed in the other parameters between the two groups (**Table 2**). Univariate and multivariate logistic regression analyses identified fasting C-peptide, HbA1c, and urine creatinine as independent risk factors for the MAFLD (**Supplementary Table S3**).

**Table 2:** Clinical parameters related to glycometabolism of enrolled T2DM patients.

Variables		Non-MAFLD (n=2231)	MAFLD (n=1074)	t/χ2/Z value	*p* value
GLU (Fasting, mmol/L)		9.20±3.57	9.31±3.37	0.836	0.403
GLU (2 hours postprandial, mmol/L)		13.60±4.89	14.261±4.88	3.670	<0.001
C peptide (Fasting, ng/mL)		1.72±1.13	2.14±1.24	9.343	<0.001
C peptide (2 hours postprandial, ng/mL)		3.59±2.56	4.21±2.79	6.115	<0.001
HbA1c (%)		9.47±2.60	9.77±2.34	3.311	0.001
Cr (mmol/L)		69.16±26.64	69.06±21.45	0.121	0.904
Microalbuminuria (mg/L)		109.10±430.24	85.67±302.50	1.807	0.071
Urine creatinine (umol/L)		11219.40±6623.04	12164.56±6741.25	3.820	<0.001
Urine protein	–	1923(86.2%)	909(84.6%)	1.011	0.312
	1+	179(8.0%)	114(10.6%)		
	2+	88(3.9%)	36(3.4%)		
	3+	41(1.8%)	15(1.4%)		
Urine sugar	–	955(42.8%)	414(38.5%)	1.513	0.130
	1+	131(5.9%)	72(6.7%)		
	2+	140(6.3%)	76(7.1%)		
	3+	359(16.1%)	201(18.7%)		
	4+	646(29.0%)	311(29.0%)		
Uroketone	–	2035(91.2%)	990(92.2%)	0.912	0.362
	1+	107(4.8%)	46(4.3%)		
	2+	65(2.9%)	22(2.1%)		
	3+	24(1.1%)	16(1.5%)		

T2DM, Type 2 diabetes mellitus; MAFLD, Metabolic associated fatty liver disease; GLU, Glucose; Cr, Creatinine; HbA1c, Glycosylated hemoglobin.

**Figure 3:**
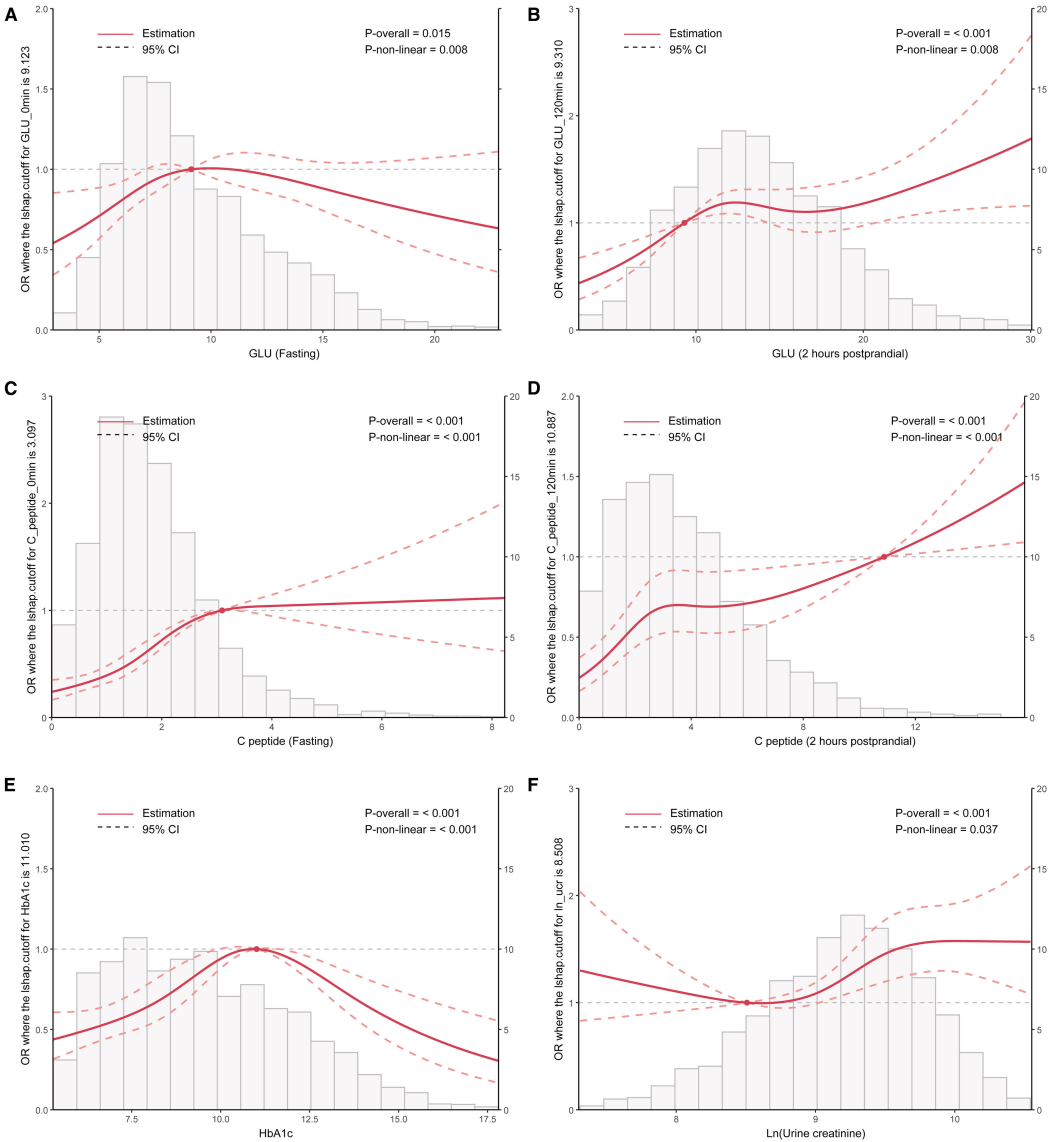
RCS curve analysis exploring the association between clinical parameters including HbA1c, Ln (urine creatinine), fasting and postprandial GLU/C-peptide levels, and the OR value of MAFLD in patients with T2DM. T2DM, Type 2 diabetes mellitus; MAFLD, Metabolic associated fatty liver disease; RCS, Restricted cubic splines; GLU, Glucose; HbA1c, Glycosylated hemoglobin; OR, Odds ratio.

The RCS curve provided an intuitive demonstration of these correlations, revealing that as the levels of postprandial GLU and C-peptide increase, the OR value for MAFLD also rises significantly. Notably, a significant positive correlation is observed between MAFLD and HbA1c (with levels below 11.0), whereas a negative correlation emerges when HbA1c levels reach or exceed 11.0 (p<0.01). Additionally, the correlation analysis conducted on log-transformed urinary creatinine suggested a significant nonlinear association with MAFLD (p<0.05) (**Figure 3**). These findings suggested that abnormal glycometabolism may elevate the risk of MAFLD among T2DM patients.

### Prediction model of MAFLD for T2DM

**Figure 4:**
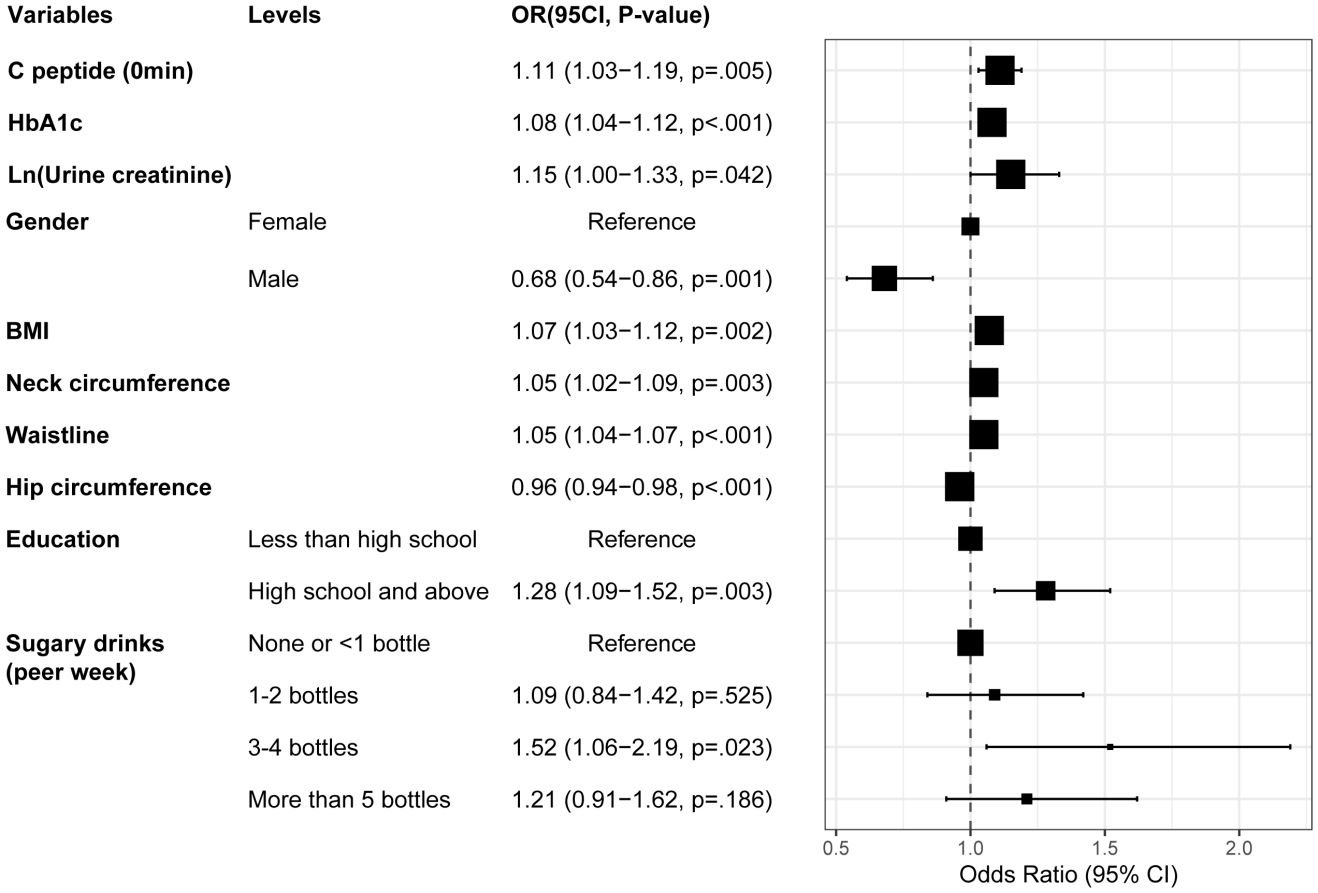
Forest plot depicting the OR values and 95% CI of independent factors for MAFLD in T2DM through multivariate logistic regression analysis. T2DM, Type 2 diabetes mellitus; MAFLD, Metabolic associated fatty liver disease; BMI, Body Mass Index; HbA1c, Glycosylated hemoglobin; OR, Odds ratio.

By pooling all parameters, a multivariate logistic regression analysis was conducted to identify the independent factors of MAFLD in patients with T2DM. These factors encompassed fasting C-peptide, HbA1c, Ln (urine creatinine), gender, BMI, neck, waist, and hip circumference, education level, and sugar drink consumption, with VIF < 5 for all variables (**Supplementary Table S4**). Subsequently, a forest plot was generated based on the OR values and corresponding 95% confidence intervals for each parameter (**Figure 4**). By integrating the regression coefficients, a MAFLD model based on glycometabolism, termed MMBG, was successfully established. Y=-7.605 + 0.106*(fasting C peptide)+ 0.080*(HbA1c)+ 0.147*Ln (urine creatinine)- 0.387*(gender, male=1,female=0) +0.066*(BMI)+ 0.052*(Waistline)+ 0.053*(neck circumference)- 0.040*(hip circumference)+ 0.255*(education, less than high school =0, high school and above=1)+ 0.089(SSB, per week, 0: None or <1 bottle, 1: 1-2bottles, 2: 3–4 bottles, 3: More than 5 bottles).

**Figure 5:**
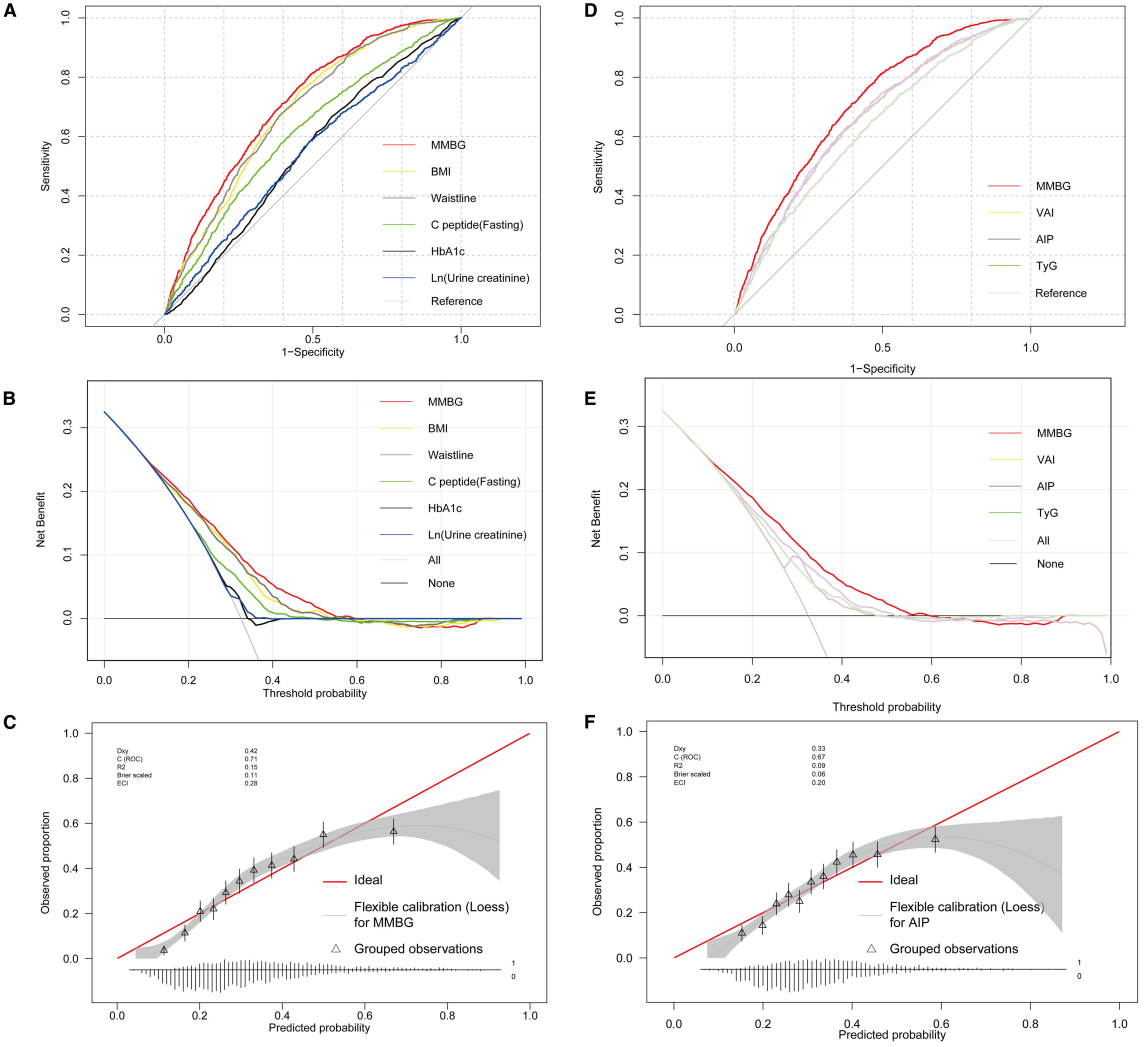
Glycometabolism-based prediction Model, MMBG, for MAFLD in T2DM. **(A, B)**, Comparative analysis of ROC and DCA curves between MMBG and clinical parameters, including BMI, waist, fasting C-peptide, HbA1c, and Ln (urine creatinine). **(C)** Analysis of the calibration curve comparing the predicted values of the MMBG model with the actual MAFLD status. **(D, E)** Comparative analysis of ROC and DCA curves between MMBG and other models, including TyG, AIP, VAI. **(F)** Analysis of the calibration curve comparing the predicted values of the AIP model with the actual MAFLD status. T2DM, Type 2 diabetes mellitus; MAFLD, Metabolic associated fatty liver disease; BMI, Body Mass Index; HbA1c, Glycosylated hemoglobin; ROC, Receiver operating characteristic; DCA, Decision Curve Analysis; VAI, Visceral Adiposity Index; TyG, Triglyceride-Glucose Index; AIP, Atherogenic Index of Plasma.

The ROC and DCA analyses were further conducted using the predicted values of the MMBG. Compared to the AUC values of each clinical indicator, such as BMI, waist circumference, C-peptide, HbA1c, and Ln (urine creatinine), the AUC value of the MMBG was significantly higher (**Figure 5A**; **Table 3**). Additionally, the DCA curve suggested that the MMBG model possessed the highest clinical application value (**Figure 5B**). Furthermore, the calibration curve visually demonstrated a high degree of fit between the MMBG and MAFLD, with a birer scaled value of only 0.11, indicating its desirability as an ideal prediction model (**Figure 5C**).

**Table 3:** Comparison of ROC curves between MMBG model and individual clinical parameters.

Variables	AUC(95%CI)	St	Sensitivity	Specificity	Z statistic (p value)
MMBG	0.712(0.696-0.728)	0.009	0.812	0.506	
BMI	0.685(0.669-0.701)	0.009	0.751	0.544	<0.001
Waistline	0.684(0.668-0.700)	0.009	0.670	0.613	<0.001
C peptide (Fasting)	0.615(0.598-0.632)	0.010	0.594	0.590	0.011
HbA1c	0.546(0.529-0.563)	0.010	0.667	0.436	<0.001
Ln (Urine creatinine)	0.546(0.528-0.563)	0.011	0.567	0.529	<0.001

T2DM, Type 2 diabetes mellitus; MAFLD, Metabolic associated fatty liver disease; MMBG, MAFLD model based on glycometabolism; BMI, Body Mass Index; HbA1c, Glycosylated hemoglobin; AUC, Areas under the ROC curves.

In addition, the MMBG model achieved an AUC of 0.712 in ROC analysis for MAFLD association, significantly outperforming the AUC values of the comparator models: VAI (0.664), AIP (0.666), and TyG (0.631) (**Figure 5D**; **Table 4**). Further decision curve analysis (DCA) confirmed the superior clinical utility of MMBG compared to other models (**Figure 5E**). Calibration curve analysis revealed Brier scores of 0.03, 0.04, and 0.06 for the VAI, AIP, and TyG models (**Figure 5F**), all significantly lower than that of the MMBG model, further underscoring its robustness as a predictive tool.

**Table 4:** Comparison of ROC curves between MMBG model and other models.

Variables	AUC(95%CI)	St	Sensitivity	Specificity	Z statistic (p value)
MMBG	0.712(0.696-0.728)	0.009	0.812	0.506	
VAI	0.664(0.648-0.680)	0.010	0.628	0.625	<0.001
AIP	0.666(0.650-0.682)	0.010	0.713	0.540	<0.001
TyG	0.631(0.614-0.648)	0.010	0.649	0.541	<0.001

T2DM, Type 2 diabetes mellitus; MAFLD, Metabolic associated fatty liver disease; MMBG, MAFLD model based on glycometabolism; VAI, Visceral Adiposity Index; TyG, Triglyceride-Glucose Index; AIP, Atherogenic Index of Plasma.

### NHANES data validation

A total of 1007 diabetic patients were enrolled in the study, comprising 853 patients with MAFLD (84.7%) and 154 patients without MAFLD (15.3%). Owing to the absence of serum C-peptide data, we resorted to serum insulin as an alternative. Consistent with the findings from our hospital’s cases, patients in the MAFLD group exhibited significantly elevated levels of BMI, waist and hip circumference, GLU, insulin, HbA1c, and urinary creatinine compared to the non-MAFLD group (p<0.01, respectively). Notably, there were no significant differences in other parameters such as age, gender, and education level between the two groups.

To assess the association between dietary differences and MAFLD, we analyzed the daily intake of energy, carbohydrates, sugars, fats, proteins, and dietary fiber among the patients. These intakes were categorized into four quartiles: Q1, Q2, Q3, and Q4. The comparison revealed that the intake of sugars, fats, and proteins was significantly higher in the MAFLD group (p<0.05, respectively). However, there were no significant differences in other parameters, including energy, carbohydrate, and dietary fiber, as well as smoking, drinking, and sleep habits, between the two groups. (**Supplementary Table S5**)

**Figure 6:**
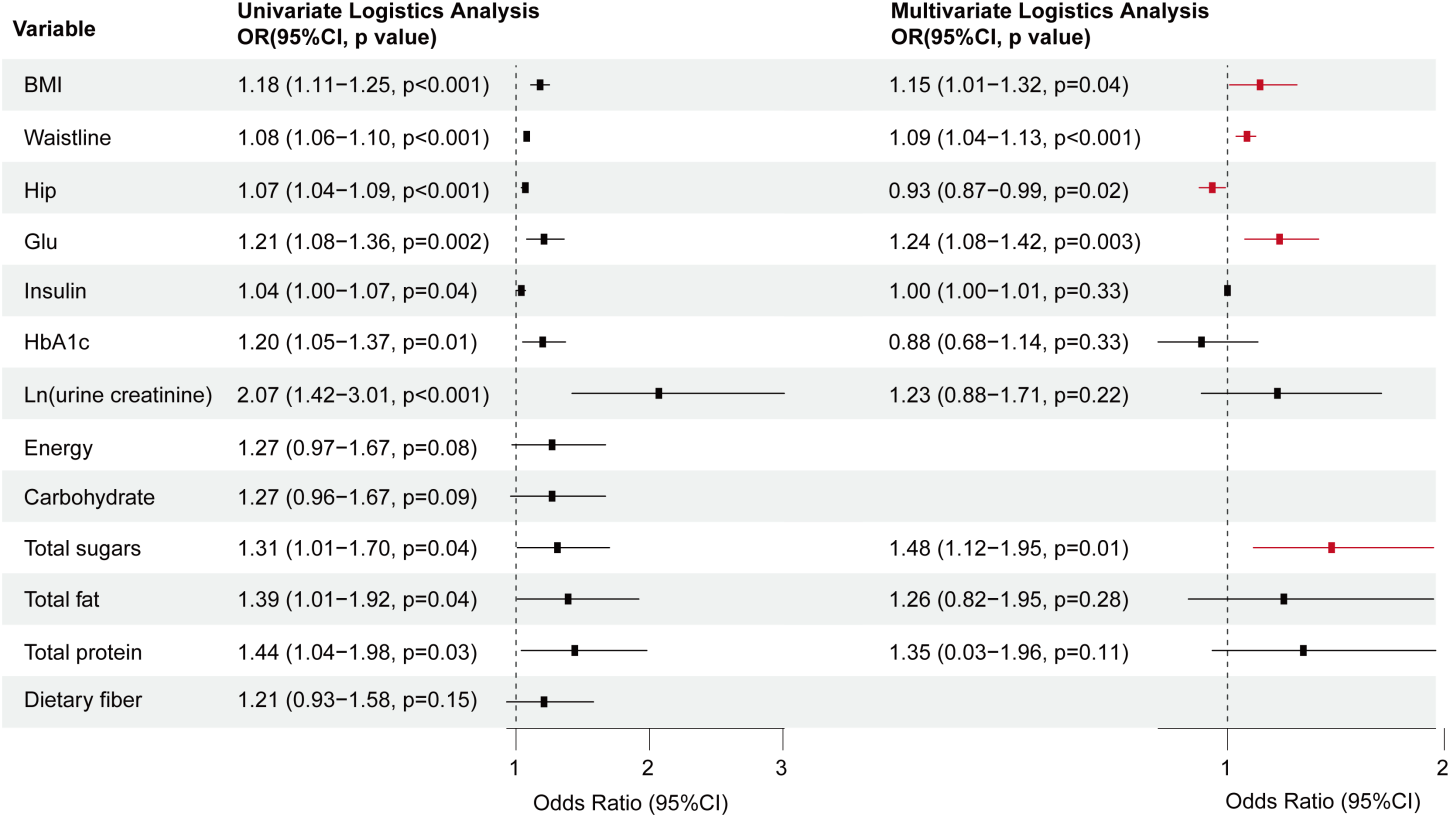
Forest plot depicting the OR values and 95% CI of independent factors for MAFLD among diabetes patients sourced from the NHANES database, analyzed through univariate and multivariate logistic regression. T2DM, Type 2 diabetes mellitus; MAFLD, Metabolic associated fatty liver disease; BMI, Body Mass Index; GLU, Glucose; HbA1c, Glycosylated hemoglobin; OR, Odds ratio.

Consistent with our findings, the univariate and multivariate logistic regression analysis revealed that BMI, waist, hip circumference, and sugars intake were independent influencing factors for the MAFLD in diabetic patients. However, a notable difference emerged in the NHANES data, where hyperglycemia emerged as an independent risk factor for MAFLD, rather than HbA1c, insulin, or urine creatinine (**Figure 6**; **Supplementary Table S6**).

## Discussion

In 2020, there was a notable paradigm shift in the classification of fatty liver diseases, marked by the introduction of MAFLD (1, 20). This revolutionary concept significantly enhanced the description of liver diseases by incorporating indicators of metabolic abnormalities, including insulin resistance, sensitivity to C-reactive protein, and various other accompanying metabolic predisposition factors (1, 20). The pathogenesis of MAFLD involves ectopic accumulation of “unhealthy” fat, primarily in the liver, muscle, and visceral adipose tissue (21). Unlike NAFLD, which excludes viral hepatitis, alcoholic liver disease, and other liver conditions, the diagnostic criteria for MAFLD adopt a more practical approach by incorporating metabolic markers (22, 23).

In this study, we observed that among T2DM patients with MAFLD, the proportion of individuals with high BMI, education and income levels was significantly higher than that among non-MAFLD. Notably, the consumption of SSB emerged as the factor for the development of MAFLD in T2DM, with an escalating risk associated with increased beverage intake. Epidemiological research across 184 countries revealed that diet-related T2DM is generally more prevalent among urban residents compared to rural ones, and individuals with higher educational levels tend to be more (24). Consistent with our findings, the odds ratio (OR) for MAFLD among T2DM patients increases significantly with elevations in serum C-peptide and GLU, indicating a close association between the abnormal glycometabolism and the risk of MAFLD.

Excessive consumption of SSB not only increases the risk of T2DM but also leads to insulin resistance, ectopic fat accumulation, and β-cell dysfunction, and involves complex interactions with conditions such as vascular–hepatic diseases. Under prolonged high-sugar stimulation, pancreatic β-cell function gradually impairs, resulting in relative or absolute insulin deficiency and leading to insulin resistance (25). In the state of insulin resistance, the liver’s sensitivity to insulin’s suppression of gluconeogenesis decreases, but hepatic *de novo* lipogenesis (DNL) is not suppressed and may even be enhanced, leading to hepatic steatosis and liver disease (26). For example, in Latino populations with the PNPLA3 GG genotype, the intake of total sugar, fructose, sucrose, and glucose was associated with liver disease incidence and liver stiffness (27). Recent evidence suggests that sugar-driven metabolic stress also affects systemic vascular damage. Chronic hyperglycemia disrupts endothelial homeostasis by inducing oxidative stress, manifested as increased p65 phosphorylation, upregulation of PTEN, and inhibition of SET protein, thereby promoting monocyte/endothelial adhesion (28). Hyperglycemia also activates the advanced glycation end products (AGEs)-RAGE axis, exacerbating oxidative stress and inflammatory phenotypic switching in vascular smooth muscle cells (29). Additionally, glycemic variability (especially high-low fluctuations) further impairs endothelial function and increases reactive oxygen species (ROS) generation via the TGF-β/SMAD3 pathway (30). In patients with familial hypercholesterolemia (FH), synergistic effects between high LDL-C levels and hyperglycemia were observed: the NLDLR group showed higher FPG and HbA1c levels than the LDLR group, suggesting that the impact of different genotypes on glucose metabolism may exacerbate vascular inflammation through mitochondrial dysfunction (31). Hyperglycemia alters monocyte glucose metabolism, promoting their recruitment into plaques and differentiation into pro-inflammatory macrophages, directly accelerating peripheral atherosclerosis (32).

Additionally, the liver-kidney axis has been increasingly recognized as playing a key role in metabolic diseases. In cases of mild acute hyperbilirubinemia (total bilirubin 12.4±7.3 mg/dL) with normal conventional renal parameters (such as creatinine and urea), patients still showed significantly elevated urinary markers of renal tubular injury (u-NGAL, u-B2M, u-OPN, u-TFF3, u-Cys), indicating the presence of subclinical renal tubular damage and supporting the important role of metabolic stress in the liver-kidney axis (33). Recent research has also confirmed the significant impact of dietary sugar intake on kidney disease. For example, a high-fructose diet (24 weeks) can induce insulin resistance, dyslipidemia, and renal dysfunction, manifested as decreased glomerular filtration rate and elevated markers of renal tubular injury (34). This damage may be related to oxidative stress and activation of inflammatory pathways resulting from fructose metabolism, in which the JNK signaling pathway plays a key role in the renal stress response (34). On the other hand, the conversion of fructose into glucose, lactate, and fatty acids in the liver may produce nephrotoxic metabolites (35). The combined intake of high-sugar and high-fat diets can accelerate functional disorders of the liver-kidney metabolic axis through insulin resistance and elevated triglyceride-rich lipoproteins (36).

In this study, we integrated the independent influencing factors of MAFLD, including fasting C-peptide, HbA1c, BMI, waist, and SSB, to develop a glycometabolism-based prediction model, MMBG, for MAFLD in T2DM. The clinical utility of MMBG was significantly superior to any individual clinical indicator, exhibiting a high degree of concordance with the actual data. Further external validation using NHANES data confirmed that BMI, waist, hip, sugar intake, and hyperglycemia were independent predictors of MAFLD in diabetic patients in the United States. Despite the high prevalence and severe implications of MAFLD in patients with T2DM, it often remains overlooked in clinical practice. Given MAFLD’s contribution to extrahepatic morbidities and mortality, it is imperative to enhance awareness among all key stakeholders, including primary care physicians, specialists, and health policymakers, regarding MAFLD as a prevalent end-organ complication of T2DM, alongside the well-established microvascular and macrovascular complications (37). As early as 2016, the European Association for the Study of the Liver jointly issued the first recommendation for universal screening of NAFLD/MAFLD in patients with T2DM (38).

Among various diagnostic tools, liver biopsy remains the gold standard for diagnosing MAFLD. However, its invasiveness poses as one of its major drawbacks. Although ultrasound offers a cost-effective option, its accuracy heavily relies on the operator’s experience and the intricacies of the technique. Other imaging modalities, including magnetic resonance spectroscopy, computed tomography, and vibration-controlled transient elastography, are prohibitively expensive for widespread screening. In this study, we demonstrate that a combination of anthropometric measurements, sugar intake assessment, and GLU/HbA1c monitoring can effectively predict the risk of MAFLD in T2DM. This approach holds significant value in assessing patient conditions and facilitating individualized interventions.

To put these findings into practice, we propose feasible strategies. Integrating SSB intake screening into routine diabetes care through electronic health record alerts and the MMBG risk model can guide personalized dietary counseling. Advocating for taxation on SSBs and subsidies for healthy alternatives, especially in high-risk urban T2DM populations, is recommended. Nutritional education for T2DM patients should emphasize the direct association between sugary beverages and T2DM-related risks, helping patients identify free and added sugars and avoid hidden sugar intake. Furthermore, the predictive value of the MMBG score can also guide early pharmacological interventions. For example, emerging lipid-lowering and hypoglycemic drugs, as well as anti-inflammatory approaches, can synergize with dietary strategies to slow the progression of MAFLD and even renal injury. Studies have shown that soy-derived genistein, in animal models, regulates lipid metabolism, reduces hepatic lipid accumulation, and indirectly improves MAFLD-related renal metabolic abnormalities by modulating gut microbiota-produced butyrate (39). Pueraria flavonoid activates the AMPK pathway, exerting lipid-lowering and anti-inflammatory effects, and may enhance drug efficacy when combined with dietary intervention (40).

As a single-center study, this paper has several defects and limitations. For example, the performance of the MMBG prediction model (AUC = 0.712) is moderate, which limits its immediate clinical application. The diagnosis of MAFLD relies on ultrasound, lacking mechanistic biomarkers such as adipokines, cytokines, or oxidative stress mediators. There is also a lack of assessment of overall dietary patterns. Future prospective studies should combine non-invasive imaging technology with biomarker analysis and further evaluate total caloric intake, macronutrients, overall dietary patterns, and physical activity levels to comprehensively assess the impact of dietary nutritional factors on metabolic diseases such as MAFLD.

In summary, for patients with T2DM, SSB is an independent risk factor for MAFLD. Based on the positive correlation between MAFLD and abnormal glycometabolism, the developed MMBG model can effectively predict the risk of MAFLD in T2DM and will have significant value in patient assessment and personalized intervention strategies.

## Data Availability

The original contributions presented in the study are included in the article/**Supplementary Material**. Further inquiries can be directed to the corresponding authors.
