# Supplementary material for: Sugar-sweetened beverage consumption predicts metabolic associated fatty liver disease in patients with type 2 diabetes mellitus
Source: Front Endocrinol (Lausanne). 2025 Oct 1;16:1651370. doi: 10.3389/fendo.2025.1651370 (PMC12520918; doi:10.3389/fendo.2025.1651370)
Supplement: Supplementary file 1 [file DataSheet1.docx]

**Sugar-Sweetened Beverage Consumption Predicts Metabolic Associated Fatty Liver Disease in Patients with Type 2 Diabetes Mellitus**

Zhenjun Yu^1#^, Mengdie Chen^2#^, Shicheng Gu^1^, Chaohui Wang^1^, Ping Feng^2^*, Gang Lin^1^*

1. Department of Gastroenterology, Taizhou Central Hospital (Taizhou University Hospital), Taizhou, Zhejiang 318000, PR China.
2. Department of Endocrinology, Taizhou Central Hospital (Taizhou University Hospital), Taizhou, Zhejiang 318000, PR China.

**Supplementary Figures**

Supplementary Figure S1: Utilization of the MICE package to demonstrate the missing data in the enrolled T2DM cases and carry out multiple imputation procedures.


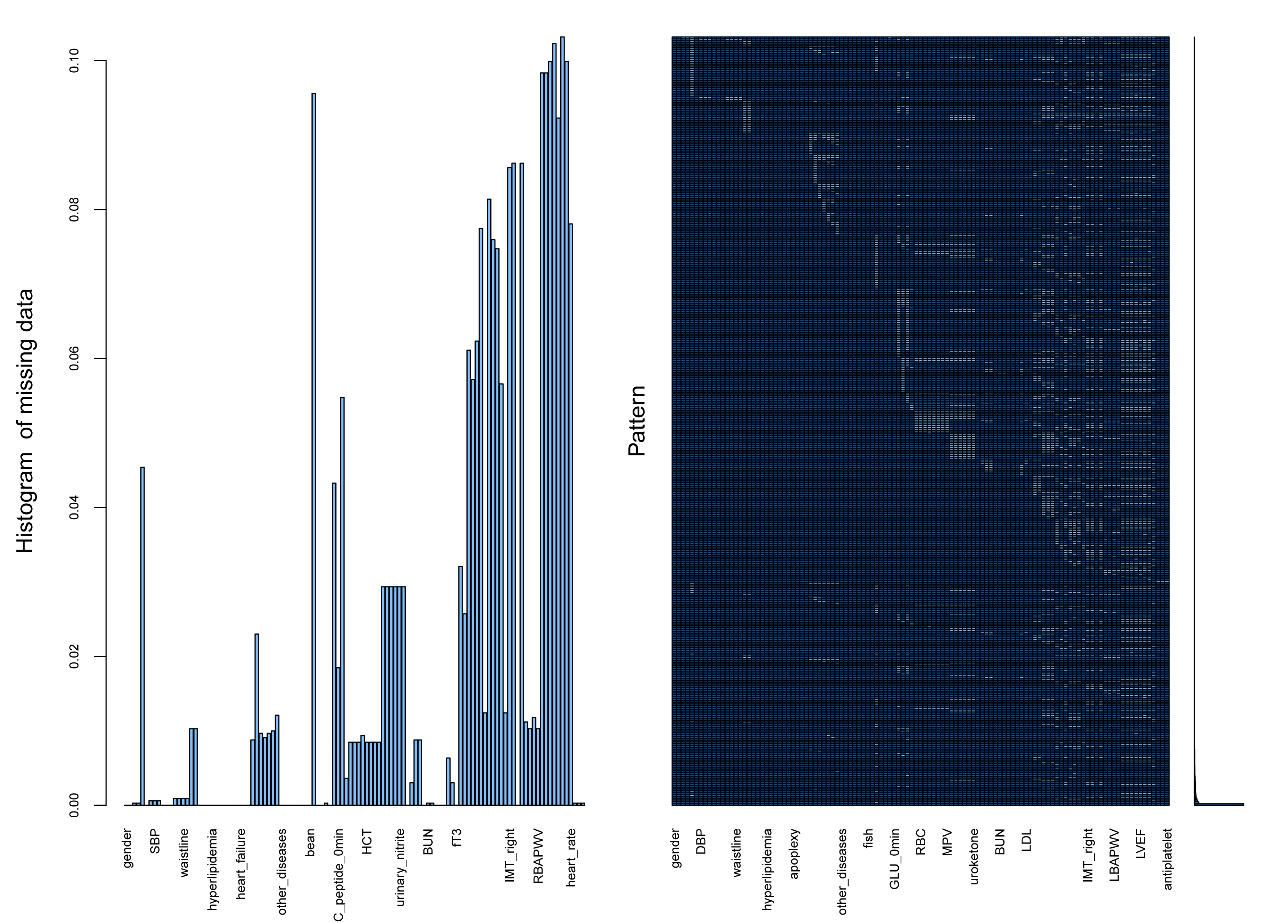


Abbreviations: T2DM, Type 2 diabetes mellitus.

Supplementary Figure S2: Forest plot depicting the OR values and 95% CI of independent factors for MAFLD in T2DM through multivariate logistic regression analysis, focusing solely on the general characteristics.


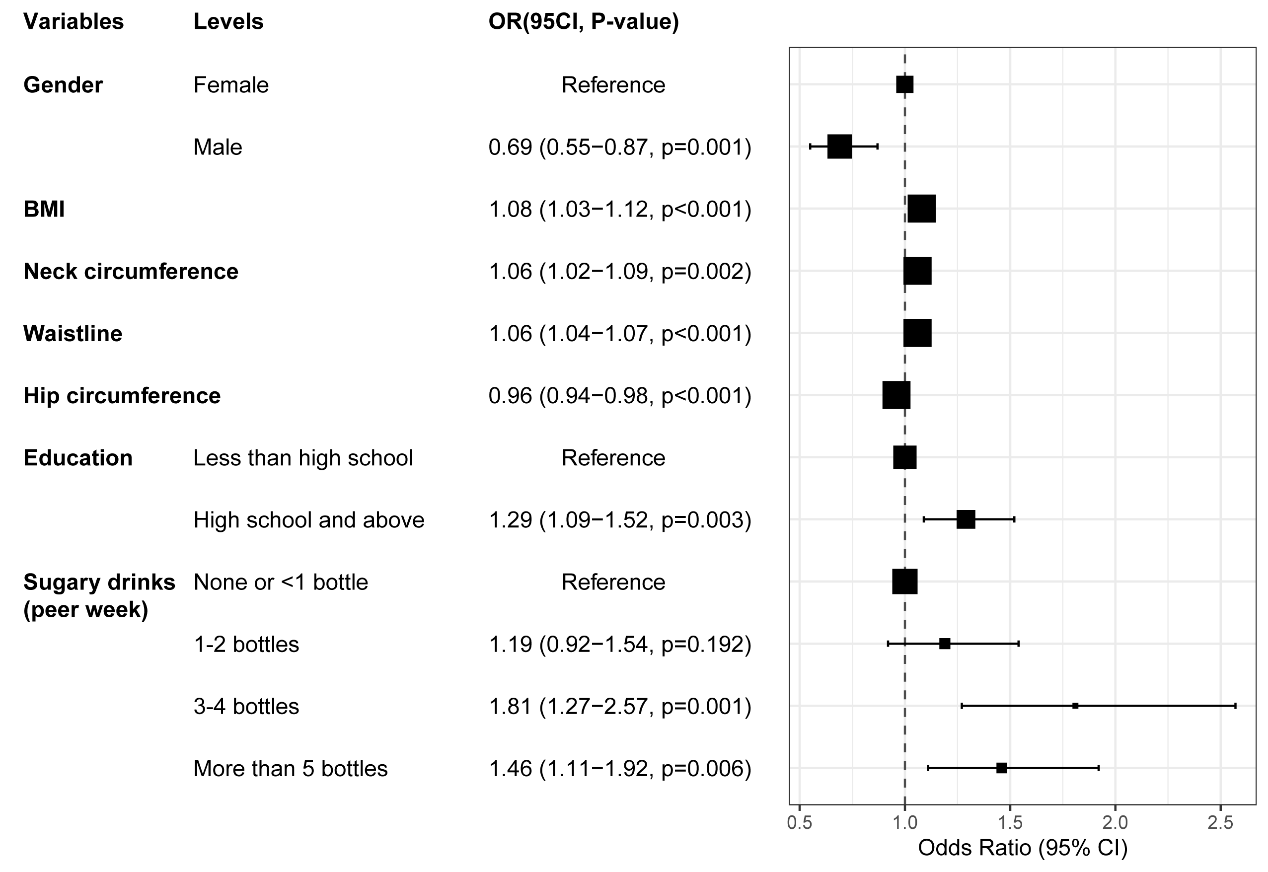


Abbreviations: T2DM, Type 2 diabetes mellitus; MAFLD, Metabolic associated fatty liver disease; BMI, Body Mass Index; OR, Odds ratio.

Supplementary Figure S3: Glycometabolism-based prediction Model, MMBG, for MAFLD in T2DM. A-B, Comparative analysis of ROC and DCA curves between MMBG and other models, including AIP, VAI and TyG. Analysis of the calibration curve comparing the predicted values of the AIP model with the actual MAFLD status.


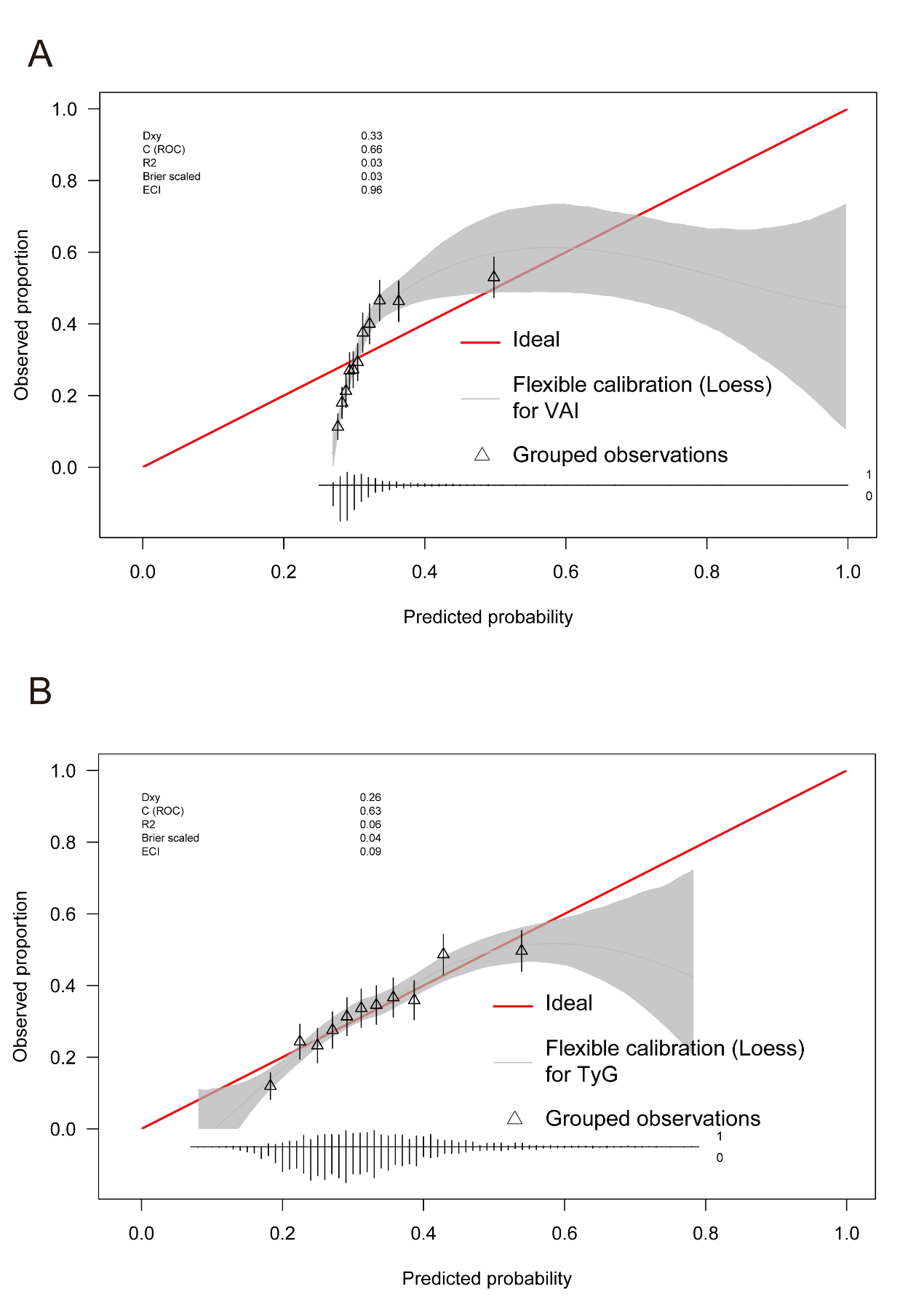


Abbreviations: T2DM, Type 2 diabetes mellitus; MAFLD, Metabolic associated fatty liver disease; VAI, Visceral Adiposity Index; TyG, Triglyceride-Glucose Index; AIP, Atherogenic Index of Plasma.

**Supplementary Tables**

Supplementary Table S1: Univariate and multivariate logistic regression analysis of general characteristics for T2DM patients with MAFLD.

| Variables | | Univariate Logistics Regression  OR（95%CI） | p value | Multivariate Logistics Regression  OR（95%CI） | p value |
| --- | --- | --- | --- | --- | --- |
| Age (years) | | 0.98 (0.97-0.98) | <0.001 | 0.99(0.98-1.00) | 0.120 |
| Gender (Male) | | 1.26 (1.07-1.48) | 0.006 | 0.69 (0.55-0.87) | 0.001 |
| BMI (kg/m^2^) | | 1.18 (1.16-1.21) | <0.001 | 1.08 (1.03-1.12) | <0.001 |
| Head circumference (cm) | | 1.16 (1.12-1.20) | <0.001 |  |  |
| Neck circumference (cm) | | 1.15 (1.13-1.18) | <0.001 | 1.06 (1.02-1.09) | 0.002 |
| Waistline (cm^2^) | | 1.07 (1.06-1.08) | <0.001 | 1.06 (1.04-1.07) | <0.001 |
| Hip circumference (cm^2^) | | 1.07 (1.06-1.08) | <0.001 | 0.96 (0.94-0.98) | <0.001 |
| Education (a high school education or higher) | | 1.56 (1.34-1.81) | <0.001 | 1.29 (1.09-1.52) | 0.003 |
| Full time job | | 1.33 (1.14-1.55) | <0.001 | 1.01(0.82-1.23) | 0.957 |
| Annual household income (￥) | <10000 |  |  |  |  |
|  | 10000-30000 | 1.15 (0.59-2.23) | 0.682 |  |  |
|  | 30000-100000 | 1.41 (0.78-2.53) | 0.251 |  |  |
|  | 100000-300000 | 1.50 (0.84-2.66) | 0.166 |  |  |
|  | >300000 | 1.73 (0.97-3.09) | 0.065 |  |  |
| Smoking | None |  |  |  |  |
|  | Infrequent | 1.18 (0.83-1.68) | 0.347 |  |  |
|  | Regular | 1.13 (0.95-1.33) | 0.161 |  |  |
| Drinking (alcohol) | None |  |  |  |  |
|  | Infrequent | 1.14 (0.97-1.33) | 0.121 |  |  |
|  | Regular | 1.11 (0.82-1.49) | 0.493 |  |  |
| Vegetables  (per day) | <200g |  |  |  |  |
|  | 200-400g | 0.82 (0.69-0.97) | 0.023 | 0.93(0.77-1.13) | 0.472 |
|  | 400-600g | 0.67 (0.55-0.83) | <0.001 | 0.90(0.72-1.13) | 0.369 |
|  | >600g | 0.48 (0.23-1.02) | 0.055 | 0.51(0.23-1.16) | 0.107 |
| Fruit  (per day) | <200g |  |  |  |  |
|  | 200-400g | 1.16 (0.98-1.37) | 0.094 | 1.09(0.91-1.31) | 0.330 |
|  | 400-600g | 1.61 (1.20-2.18) | 0.002 | 1.46(1.01-2.02) | 0.051 |
|  | >600g | 1.43 (0.76-2.69) | 0.271 | 1.16(0.57-2.37) | 0.684 |
| Fish  (per day) | <1 time |  |  |  |  |
|  | >2 times, <100g/time | 0.94 (0.74-1.20) | 0.618 |  |  |
|  | >2 times，  >100g/time | 0.92 (0.76-1.10) | 0.362 |  |  |
| Bean  (per day) | <100g |  |  |  |  |
|  | 100-250g | 0.90 (0.77-1.05) | 0.196 |  |  |
|  | 250-400g | 1.25 (0.86-1.82) | 0.241 |  |  |
|  | >400g | 1.20 (0.60-2.39 | 0.612 |  |  |
| Salt  (per day) | <4g |  |  |  |  |
|  | 4-6g | 1.17 (0.81-1.70) | 0.400 |  |  |
|  | 6-8g | 1.18 (0.82-1.69) | 0.368 |  |  |
|  | >8g | 1.26 (0.86-1.84) | 0.237 |  |  |
| Sugary drinks  (per week) | None or < 1 bottle |  |  |  |  |
|  | 1-2 bottles | 1.50 (1.18-1.91) | 0.001 | 1.19 (0.92-1.54) | 0.192 |
|  | 3-4 bottles | 2.16 (1.55-3.01,) | <0.001 | 1.81 (1.27-2.57) | 0.001 |
|  | More than 5 bottles | 2.06 (1.61-2.64) | <0.001 | 1.46 (1.11-1.92) | 0.006 |
| Sleep | Good |  |  |  |  |
|  | Poor | 1.12 (0.94-1.34) | 0.208 |  |  |
|  | Drug dependence | 1.06 (0.32-3.54) | 0.920 |  |  |

Abbreviations: T2DM, Type 2 diabetes mellitus; MAFLD, Metabolic associated fatty liver disease; BMI, Body Mass Index.

Supplementary Table S2: The multi-model logistic regression analysis for MAFLD exploring consumption of sugary drinks in T2DM.

| Variables | | B | SE | Wald | p value | OR (95%CI) |
| --- | --- | --- | --- | --- | --- | --- |
| Model1 | | | | | | |
| Sugary drinks  (per week) | None or < 1 bottle | Reference | | | | |
|  | 1-2 bottles | 0.41 | 0.12 | 3.28 | 0.001 | 1.50(1.18-1.91) |
|  | 3-4 bottles | 0.77 | 0.17 | 4.56 | <0.001 | 2.16(1.55-3.01) |
|  | More than 5 bottles | 0.72 | 0.13 | 5.73 | <0.001 | 2.06(1.61-2.64) |
| p value for trend | | <0.001 | | | | |
| Model2 (adjust age, gender) | | | | | | |
| Sugary drinks  (per week) | None or < 1 bottle | Reference | | | | |
|  | 1-2 bottles | 0.24 | 0.13 | 1.89 | 0.059 | 1.28(0.99-1.64) |
|  | 3-4 bottles | 0.56 | 0.18 | 3.21 | 0.001 | 1.76(1.24-2.47) |
|  | More than 5 bottles | 0.49 | 0.14 | 3.64 | <0.001 | 1.64(1.26-2.14) |
| p value for trend | | <0.001 | | | | |
| Model3 (adjust age, gender, BMI, waistline, education) | | | | | | |
| Sugary drinks  (per week) | None or < 1 bottle | Reference | | | | |
|  | 1-2 bottles | 0.15 | 0.14 | 1.1 | 0.276 | 1.16(0.89-1.52) |
|  | 3-4 bottles | 0.58 | 0.19 | 3.14 | 0.002 | 1.79(1.25-2.58) |
|  | More than 5 bottles | 0.34 | 0.14 | 2.38 | 0.017 | 1.41(1.06-1.87) |
| p value for trend | | <0.001 | | | | |
| Model4 (adjust all variables) | | | | | | |
| Sugary drinks  (per week) | None or < 1 bottle | Reference | | | | |
|  | 1-2 bottles | 0.12 | 0.14 | 0.85 | 0.396 | 1.13(0.86-1.48) |
|  | 3-4 bottles | 0.54 | 0.19 | 2.88 | 0.004 | 1.72(1.19-2.50) |
|  | More than 5 bottles | 0.27 | 0.15 | 1.81 | 0.070 | 1.31 |
| p value for trend | | <0.001 | | | | |

Abbreviations: T2DM, Type 2 diabetes mellitus; MAFLD, Metabolic associated fatty liver disease; BMI, Body Mass Index; OR, Odds ratio.

Supplementary Table S3: Univariate and multivariate logistic regression analysis of clinical parameters for T2DM patients with MAFLD.

| Variables | | Univariate Logistics Regression  OR（95%CI） | p value | Multivariate Logistics Regression  OR（95%CI） | p value |
| --- | --- | --- | --- | --- | --- |
| GLU (Fasting, mmol/L) | | 1.01 (0.99-1.03) | 0.403 |  |  |
| GLU (2 hours postprandial, mmol/L) | | 1.03 (1.01-1.04) | <0.001 | 1.01 (0.99-1.03) | 0.180 |
| C peptide (Fasting, ng/mL) | | 1.34 (1.26-1.43) | <0.001 | 1.34 (1.22-1.46) | <0.001 |
| C peptide (2 hours postprandial, ng/mL) | | 1.09 (1.06-1.12) | <0.001 | 1.04 (1.00-1.09) | 0.058 |
| HbA1c (%) | | 1.05 (1.02-1.08) | 0.002 | 1.11 (1.07-1.15) | <0.001 |
| Cr (mmol/L) | | 1.00 (1.00-1.00) | 0.910 |  |  |
| Microalbuminuria (mg/L) | | 1.00 (1.00-1.00) | 0.114 |  |  |
| Ln (urine creatinine) | | 1.27 (1.13-1.44) | <0.001 | 1.24 (1.08-1.41) | 0.002 |
| Urine protein | - |  |  |  |  |
|  | 1+ | 1.35 (1.05-1.73) | 0.018 | 1.09 (0.84-1.41) | 0.523 |
|  | 2+ | 0.87 (0.58-1.29) | 0.474 | 0.67 (0.44-1.02) | 0.063 |
|  | 3+ | 0.77 (0.43-1.41) | 0.400 | 0.60 (0.32-1.12) | 0.109 |
| Urine sugar | - |  |  |  |  |
|  | 1+ | 1.27 (0.93-1.73) | 0.133 | 1.19 (0.86-1.65) | 0.281 |
|  | 2+ | 1.25 (0.93-1.69) | 0.144 | 1.18 (0.86-1.62) | 0.304 |
|  | 3+ | 1.29 (1.05-1.59) | 0.016 | 1.15 (0.92-1.45) | 0.212 |
|  | 4+ | 1.11 (0.93-1.33) | 0.248 | 1.09 (0.89-1.34) | 0.389 |
| Uroketone | - |  |  |  |  |
|  | 1+ | 0.88 (0.62-1.26) | 0.493 |  |  |
|  | 2+ | 0.70 (0.43-1.13) | 0.146 |  |  |
|  | 3+ | 1.37 (0.72-2.59) | 0.332 |  |  |

Abbreviations: T2DM, Type 2 diabetes mellitus; MAFLD, Metabolic associated fatty liver disease; GLU, Glucose; Cr, Creatinine; HbA1c, Glycosylated hemoglobin; OR, Odds ratio.

Supplementary Table S4: VIF value of individual clinical parameters in multicollinearity analysis of MAFLD.

| Variables | VIF value |
| --- | --- |
| Gender | 1.780 |
| Education | 1.108 |
| BMI | 4.389 |
| Neck_circumference | 2.897 |
| Waistline | 4.670 |
| Hip_circumference | 3.679 |
| Sugary_drinks | 1.231 |
| C_peptide_0min | 1.359 |
| HbA1c | 1.283 |
| Ln_ucr | 1.156 |

Abbreviations: T2DM, Type 2 diabetes mellitus; MAFLD, Metabolic associated fatty liver disease; BMI, Body Mass Index; VIF, Variance Inflation Factor.

Supplementary Table S5: Clinical characteristics of enrolled patients with diabetes from NhANES data.

| Variables | | | Non-MAFLD  (n=154) | MAFLD  (n=853) | *p* value |
| --- | --- | --- | --- | --- | --- |
| Age (years) | | | 59.78±1.51 | 58.49±0.98 | 0.48 |
| Gender (Male) | | | 76(48.8%) | 459(54.4%) | 0.51 |
| BMI (kg/m^2^) | | | 27.93±0.74 | 34.85±0.55 | < 0.001 |
| Waistline (cm^2^) | | | 98.01±1.72 | 115.22±1.04 | < 0.001 |
| Hip circumference (cm^2^) | | | 103.62±1.61 | 116.43±1.03 | < 0.001 |
| Education (a high school education or higher) | | | 132(92.8%) | 744(93.4%) | 0.80 |
| GLU (mmol/L) | | | 8.00±0.22 | 9.40±0.18 | < 0.001 |
| Insulin (uIU/mL) | | | 15.07±1.67 | 27.65±2.08 | < 0.001 |
| HbA1c (%) | | | 7.04±0.09 | 7.41±0.09 | 0.003 |
| Cr (mmol/ml) | | | 88.16±4.46 | 77.70±1.23 | 0.05 |
| Microalbuminuria (mg/L) | | | 120.56±25.45 | 112.09±18.14 | 0.78 |
| Urine creatinine (umol/L) | | | 9425.83±534.17 | 12162.16±358.66 | < 0.001 |
| Smoking | None | | 44(66.4%) | 298(74.9%) | 0.35 |
|  | Infrequent | | 5(2.4%) | 22(3.3%) |  |
|  | Regular | | 24(31.2%) | 92(21.8%) |  |
| Drinking (alcohol) | None | | 17(7.6%) | 74(10.0%) | 0.53 |
|  | Infrequent | | 113(79.4%) | 589(72.9%) |  |
|  | Regular | | 22(13.0%) | 159(17.1%) |  |
| Energy  (kcal per day) | | Q1 | 48(28.8%) | 205(22.5%) | 0.06 |
|  |  | Q2 | 39(25.6%) | 213(21.9%) |  |
|  |  | Q3 | 35(30.3%) | 215(26.3%) |  |
|  |  | Q4 | 32(15.3%) | 220(29.3%) |  |
| Carbohydrate  (g per day) | | Q1 | 43(28.7%) | 209(20.4%) | 0.09 |
|  |  | Q2 | 39(25.4%) | 213(25.6%) |  |
|  |  | Q3 | 42(28.7%) | 209(24.3%) |  |
|  |  | Q4 | 30(17.2%) | 222(29.7%) |  |
| Total sugars  (g per day) | | Q1 | 31(27.7%) | 221(21.6%) | 0.03 |
|  |  | Q2 | 55(30.9%) | 198(23.0%) |  |
|  |  | Q3 | 41(24.2%) | 209(24.6%) |  |
|  |  | Q4 | 27(17.2%) | 225(30.9%) |  |
| Total fat (g per day) | | Q1 | 55(31.0%) | 197(21.2%) | 0.03 |
|  |  | Q2 | 37(31.0%) | 216(22.3%) |  |
|  |  | Q3 | 31(21.4%) | 219(27.6%) |  |
|  |  | Q4 | 31(16.6%) | 221(28.9%) |  |
| Total protein  (g per day) | | Q1 | 57(38.7%) | 195(19.7%) | 0.03 |
|  |  | Q2 | 34(16.2%) | 219(22.8%) |  |
|  |  | Q3 | 30(27.7%) | 221(26.3%) |  |
|  |  | Q4 | 33(17.4%) | 218(31.2%) |  |
| Dietary fiber  (g per day) | | Q1 | 43(30.1%) | 210(23.5%) | 0.13 |
|  |  | Q2 | 40(25.3%) | 211(21.7%) |  |
|  |  | Q3 | 35(26.0%) | 216(29.6%) |  |
|  |  | Q4 | 36(18.6%) | 216(25.2%) |  |
| Sleep trouble | | None | 89(51.5%) | 459(53.9%) | 0.34 |
|  |  | Infrequent | 39(20.2%) | 217(25.3%) |  |
|  |  | Regular | 26(28.4%) | 177(20.8%) |  |

Abbreviations: MAFLD, Metabolic associated fatty liver disease; GLU, Glucose; Cr, Creatinine; HbA1c, Glycosylated hemoglobin.

Supplementary Table S6: Univariate and multivariate logistic regression analysis of clinical parameters for diabetes patients with MAFLD derived from NHANES data.

| Variables | Univariate Logistics Regression  OR（95%CI） | p value | Multivariate Logistics Regression  OR（95%CI） | p value |
| --- | --- | --- | --- | --- |
| BMI (kg/m^2^) | 1.18 (1.11-1.25) | <0.001 | 1.15(1.01,1.32) | 0.04 |
| Waistline (cm^2^) | 1.08 (1.06-1.10) | <0.001 | 1.09(1.04,1.13) | <0.001 |
| Hip circumference (cm^2^) | 1.07 (1.04-1.09) | <0.001 | 0.93(0.87,0.99) | 0.02 |
| GLU (mmol/L) | 1.21(1.08,1.36) | 0.002 | 1.24(1.08,1.42) | 0.003 |
| Insulin (uIU/mL) | 1.04(1.00,1.07) | 0.04 | 1.00(1.00,1.01) | 0.33 |
| HbA1c (%) | 1.20(1.05,1.37) | 0.01 | 0.88(0.68,1.14) | 0.33 |
| Ln (urine creatinine) | 2.07(1.42,3.01) | <0.001 | 1.23(0.88,1.71) | 0.22 |
| Energy | 1.27(0.97,1.67) | 0.08 |  |  |
| Carbohydrate | 1.27(0.96,1.67) | 0.09 |  |  |
| Total sugars | 1.31(1.01,1.70) | 0.04 | 1.48(1.12,1.95) | 0.01 |
| Total fat | 1.39(1.01,1.92) | 0.04 | 1.26(0.82,1.95) | 0.28 |
| Total protein | 1.44(1.04,1.98) | 0.03 | 1.35(0.93,1.96) | 0.11 |
| Dietary fiber | 1.21(0.93,1.58) | 0.15 |  |  |

Abbreviations: MAFLD, Metabolic associated fatty liver disease; GLU, Glucose; Cr, Creatinine; HbA1c, Glycosylated hemoglobin; OR, Odds ratio.
